# Supplementary material for: Whole-exome sequencing reveals the origin and evolution of hepato-cholangiocarcinoma
Source: Nat Commun. 2018 Mar 1;9:894. doi: 10.1038/s41467-018-03276-y (PMC5832792; doi:10.1038/s41467-018-03276-y)
Supplement: Supplementary file 1 — Supplementary Information [file 41467_2018_3276_MOESM1_ESM.pdf]

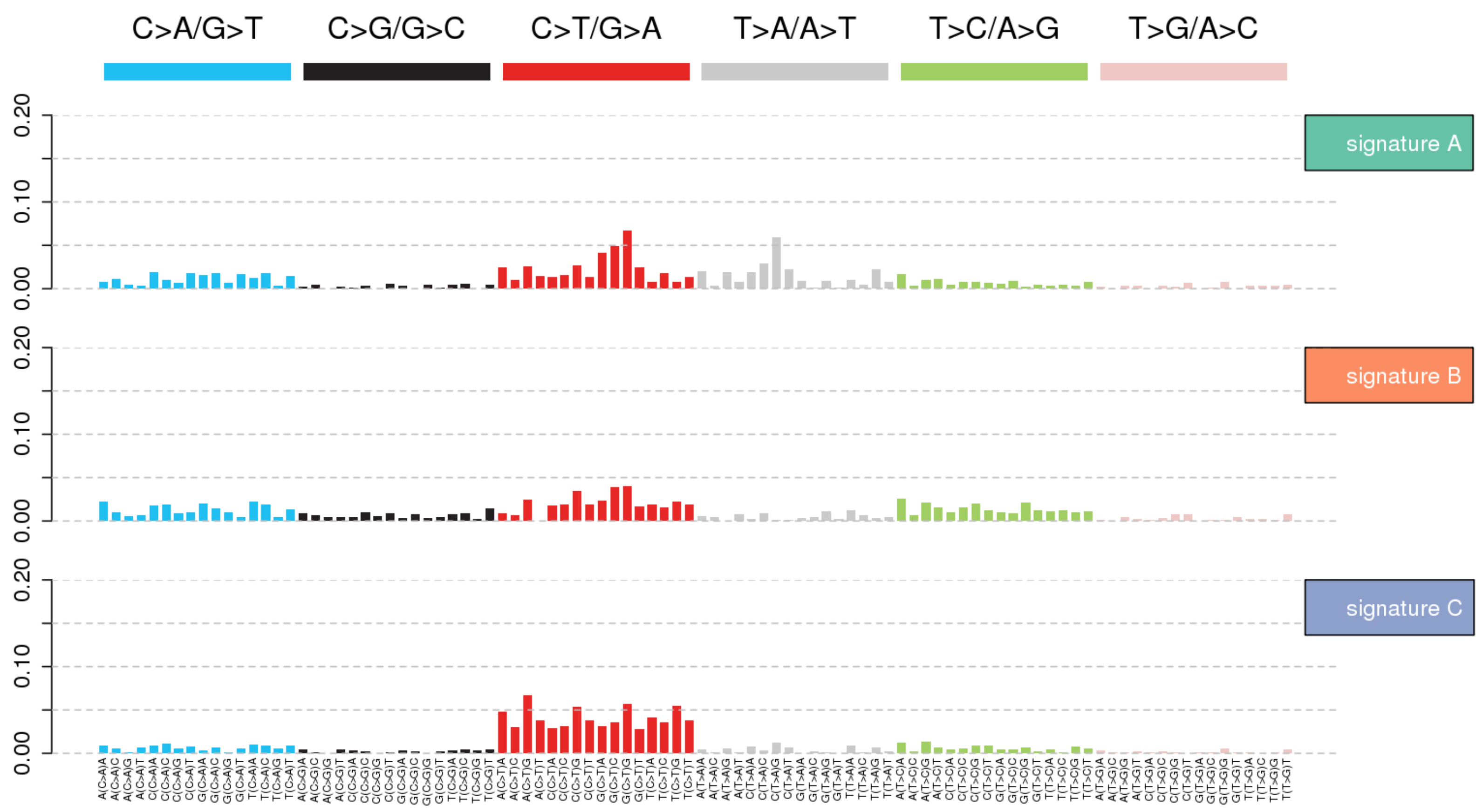

Supplementary Figure 1: mutation signatures of all tumor samples

Legend: The chart shows three mutation signatures of all tumor samples. X-axis represents 96 mutation types. Signature A, signature B and signature C indicate three different mutation signatures.

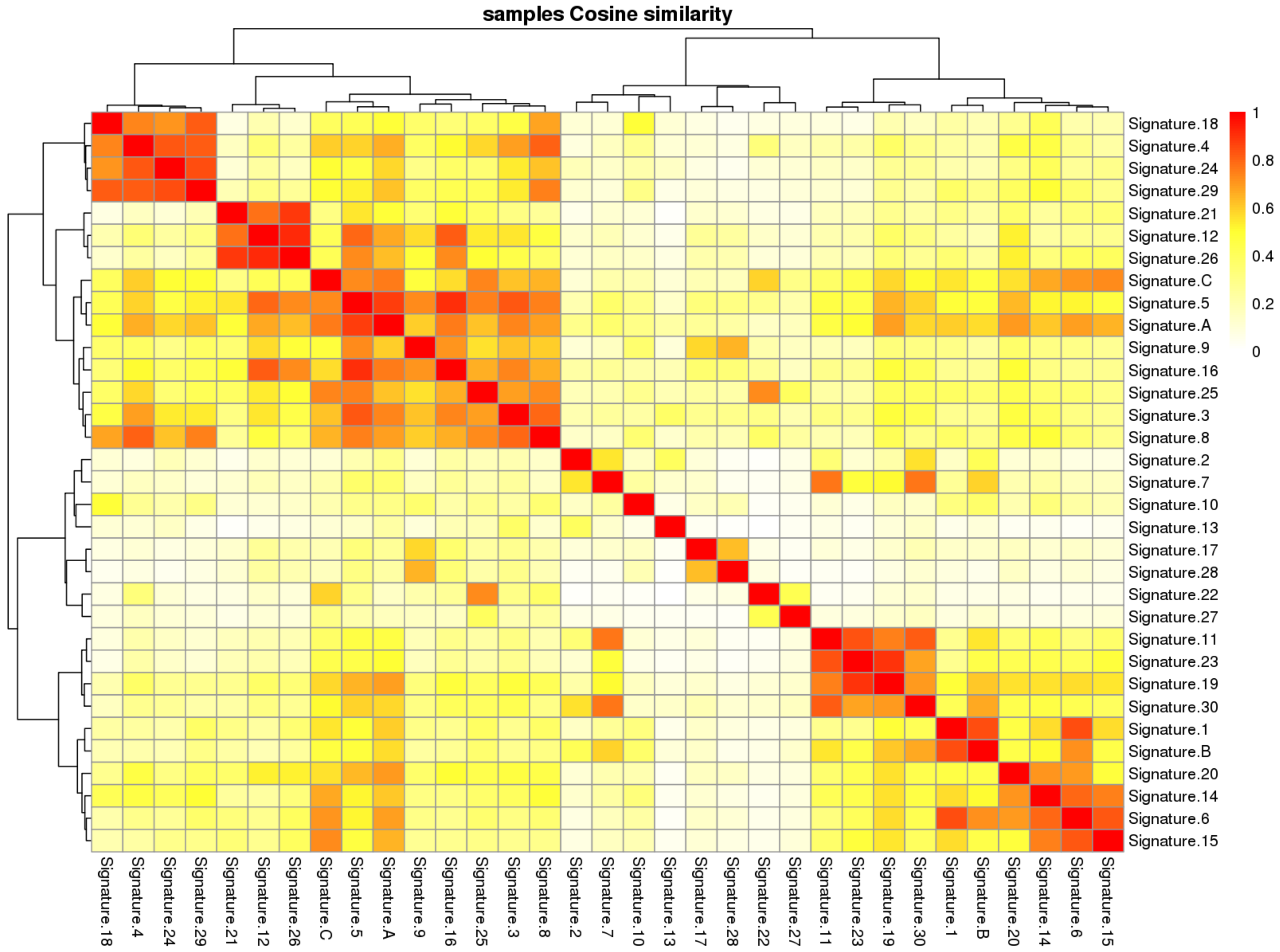

Supplementary Figure 2: Cosine similarity heatplot of identified signatures and known signatures

Legend: The plot indicates the cosine similarity of signature A, B, C and 30 known signatures. Color from white to red represents different similarity from low to high. The letter and sequential number represent identified signature and known signature respectively.

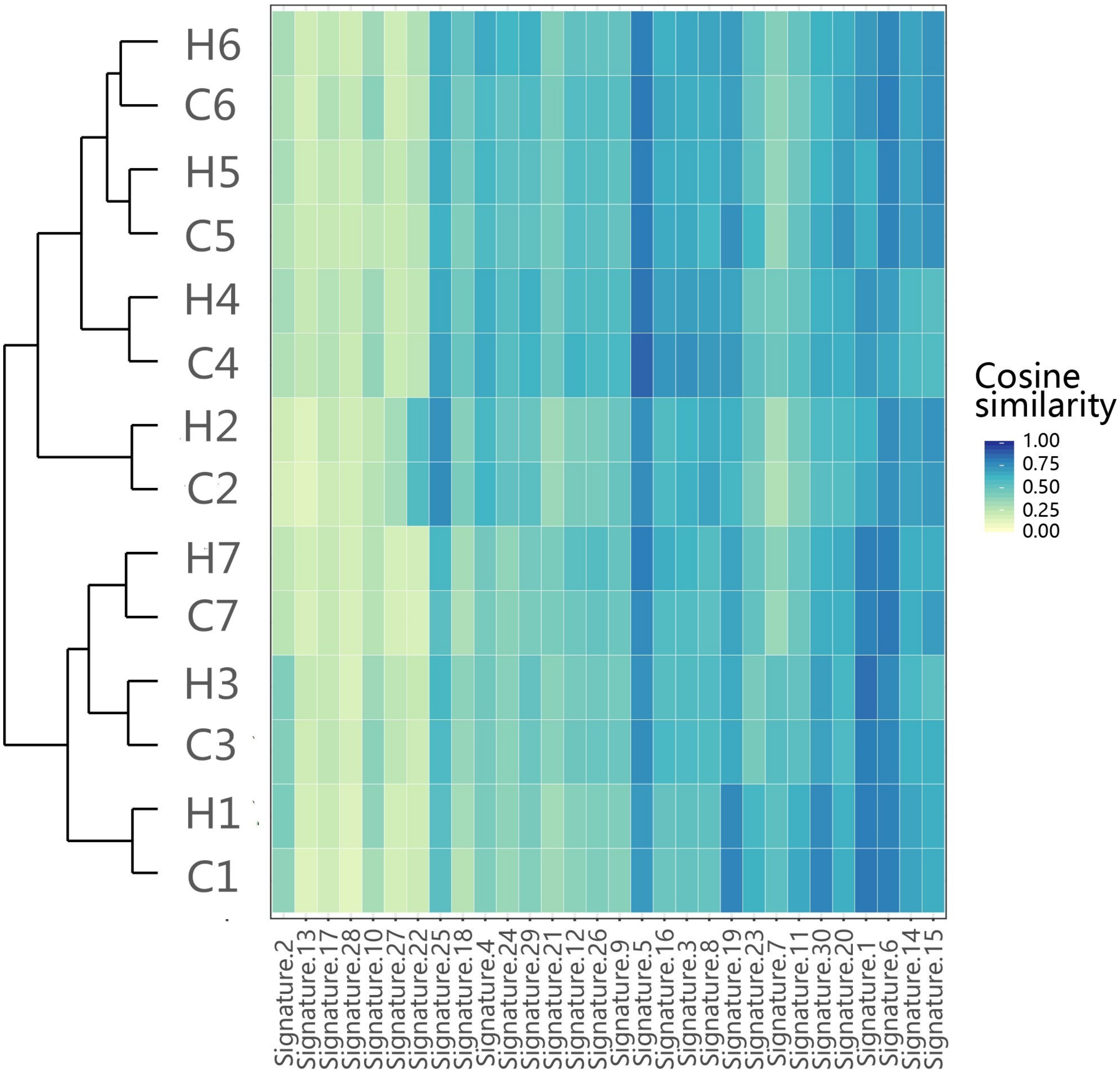

Supplementary Figure 3: The similarity between each mutational profile and each COSMIC signature

Legend: The X-axis represent the 30 known signatures and the Y-axis represent different tumor samples.

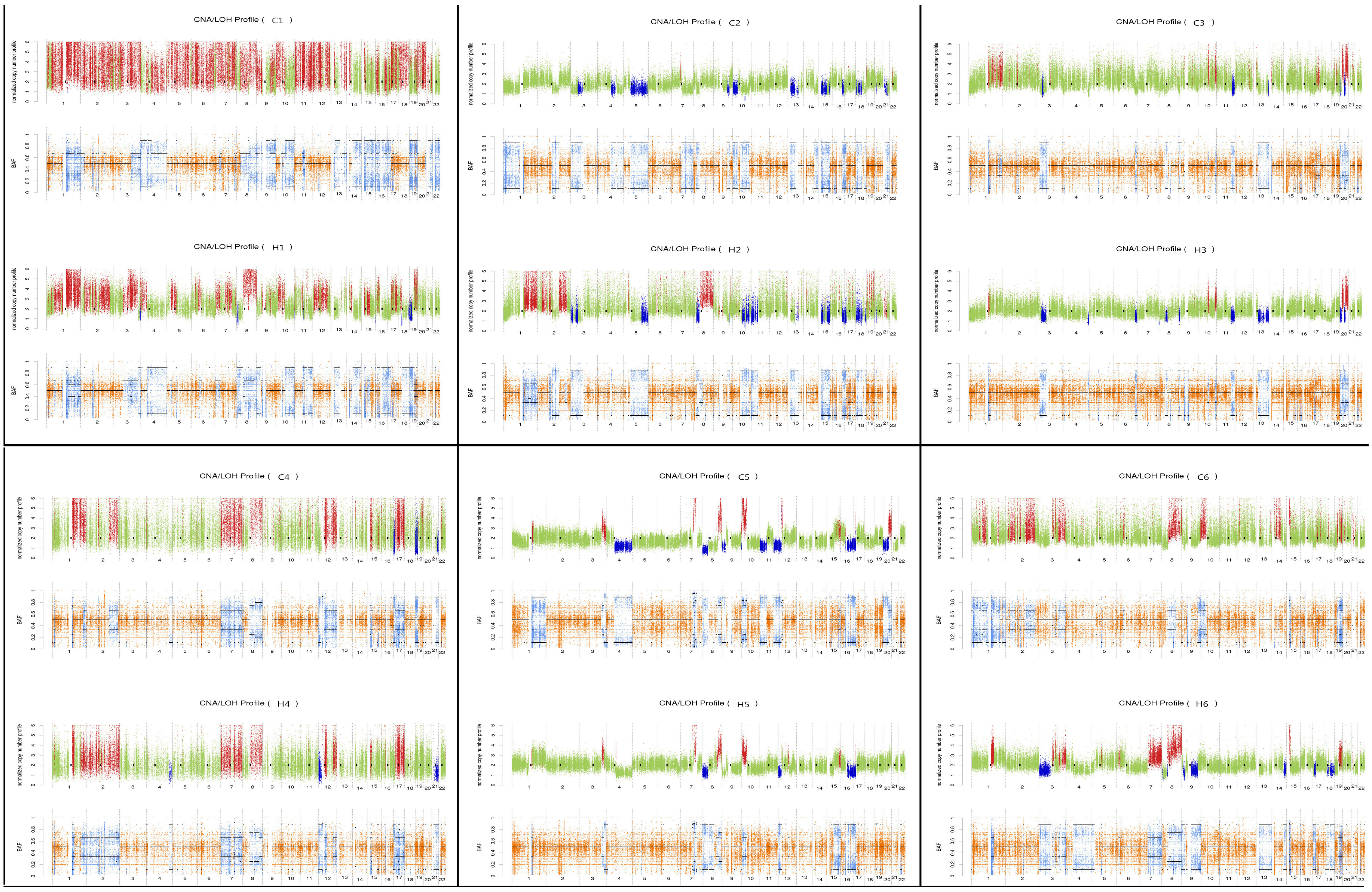

Supplementary Figure 4: Profile of somatic copy number variation on exome data

Legend: The upper chart depicts the distribution of somatic copy number variation (CNV). Red is for CNV gain, green for normal CNV and blue for CNV loss. The below chart depicts distribution of B allele frequency (BAF). Orange represents consistent distribution of allele and blue indicates loss of heterozygosity (LOH)

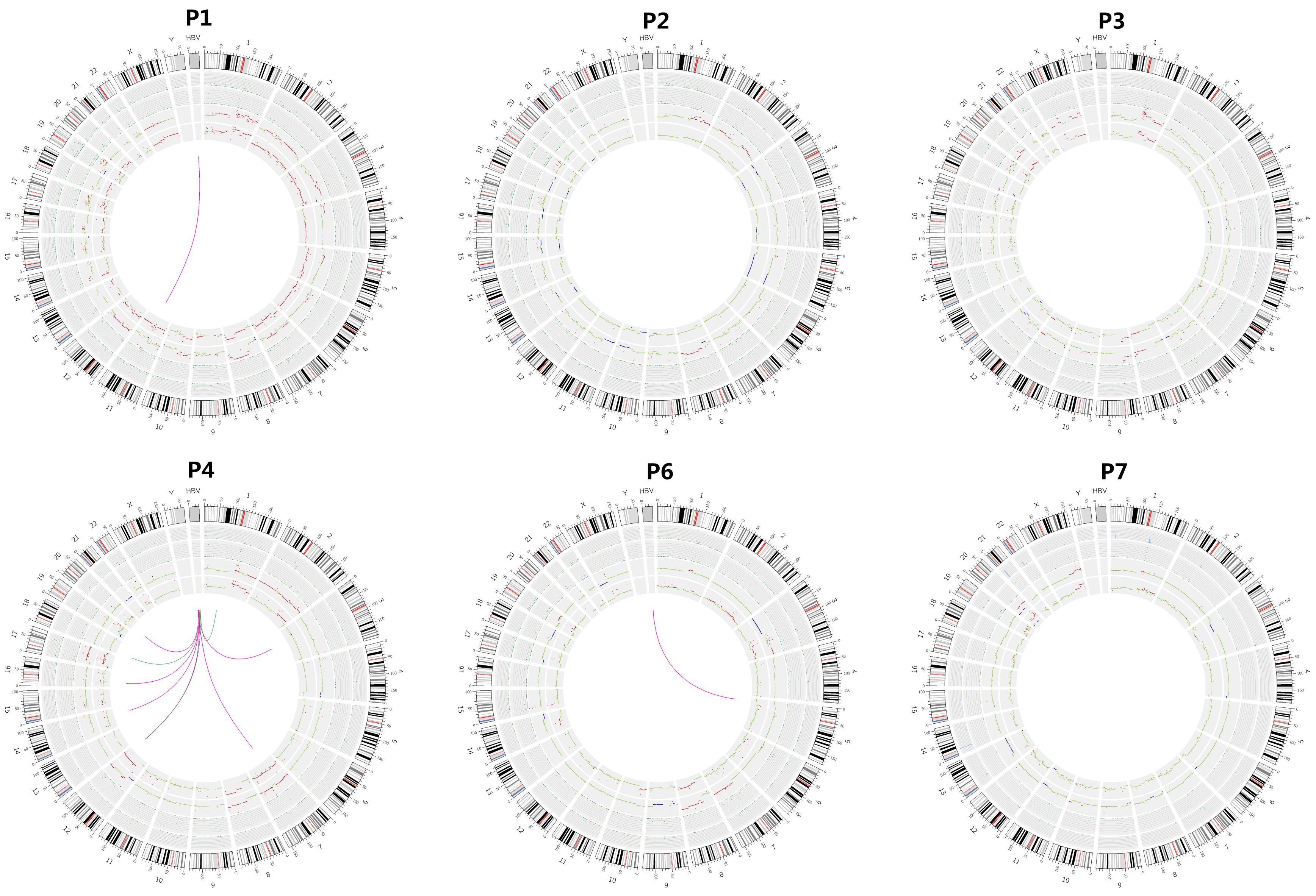

Supplementary Figure 5: Clonal relationship between HCC and iCCA components

Legend: Circos plot depicts the relationship between HCC and iCCA components on the terms of somatic nucleotide variant (SNV), somatic insertions and deletions, somatic copy number variation (CNV) and HBV integration. The first and second circles represent CNV for iCCA and HCC. Red indicates CNV gain, green normal CNV and blue CNV loss. The third and fourth circles represent SNV and indel for iCCA and HCC. Green dot indicates SNV and indel. Red curve indicates HBV integration sites for HCC.

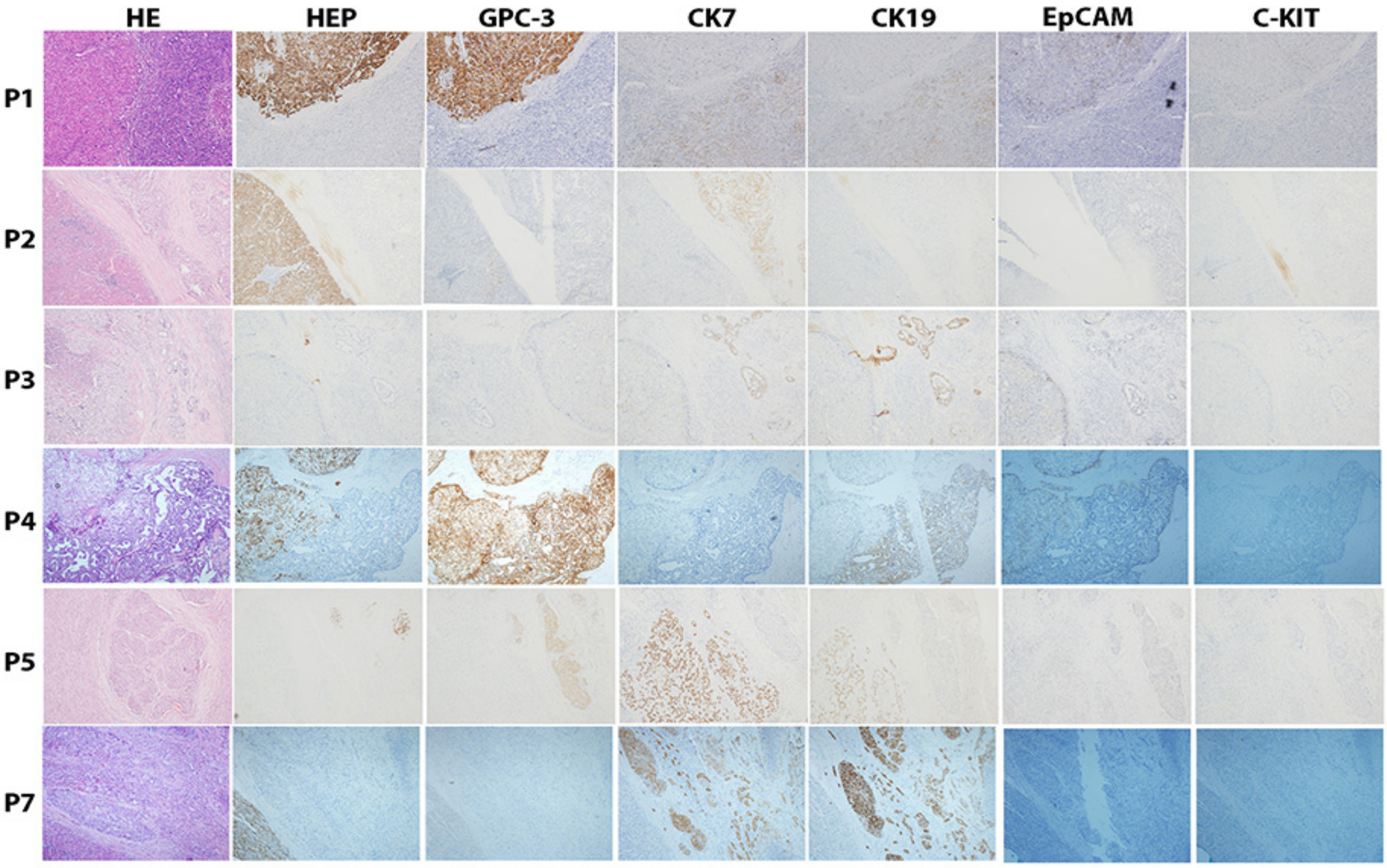

Supplementary Figure 6: the pathological features of H-ChC samples

Supplementary Table 1: Immunohistochemical profiles of liver cancer

	Hep	GPC-3	Hep/GPC-3	CK7	CK19	CK7/CK19	EpCAM	c-kit
H-ChC(n=15)	8	9	12	12	12	14	13	0
HCC (n=32)	28	21	30	6	3	8	7	0
iCCA (n=28)	2	2	4	26	27	27	20	1

Supplementary Table 2: Information of whole exome sequencing of 21 samples in 7 H-ChC patients

Sample	Average sequencing depth on target	Coverage of target region	fraction of target covered with at least 20x
N1	135.34	99.90%	99.50%
C1	152.01	99.90%	99.50%
H1	135.09	99.90%	99.60%
N2	139.83	99.90%	99.20%
C2	116.97	99.90%	98.80%
H2	96.2	99.90%	98.80%
N3	93.01	99.90%	98.90%
C3	135.16	99.90%	98.20%
H3	166.2	99.80%	98.30%
N4	107.33	99.90%	98%
C4	155.12	99.90%	99.30%
H4	139.39	99.80%	98.30%
N5	123.44	99.90%	99.50%
C5	150.44	99.90%	99.50%
H5	129.71	99.90%	99.40%
N6	144.47	99.90%	99.20%
C6	137.81	99.90%	99.60%
H6	178.73	99.90%	99.60%
N7	109.06	99.60%	99%
C7	118.97	99.60%	99.10%
H7	215.6	99.70%	99.50%

Supplementary Table 3: the HBV integration of tumor components

Sample	Flag	chr	site	read sequence
H1	161	11	73830175	AAACTCCTGGGCTCAAGTGATCTGCCCATCTCAGCCTCCCAAAGTGCTGGAATTACAGGCATGAGCCACCGTACCCAGCCAAGACTACTTTTTGTCACCATTCTATACTACAAATGATTCATTCTGCTTTTAATCTTGCTAAAATGAACT
	161	11	73830178	GAGCTCCTGGGCTCAAGTGATCTGCCCATCTCAGCCCCCCACAGTGCTGGAATTAGAGGCAAGAGCCACCGTACCCAGCCAAGACTACTTTTTGTCACCATTCTATACTACAAATGATTCATTCTGCTTTTAATCTTGCTAAAATGAACT
C4	161	1	87262301	CAGCCTAGGAACTCTTTTTTAACTCTTTTTTCTATGATAAAATATCCAGATTTGATTCTTCATCAAAGGACCTTTCCTCACAATTAGTCAACTGCAAAGTACTCAGAGCACAAGGCAGCTGCTTTTCTAAAAGAAATCTCTATTTCCAGA
	129	18	9255787	TTGAAAAAGAAAAGAAGATAAAACATGAGCATAAGTCAGAAAAAGACAAATTAGATCTTA

				GTGAATGTGTTGATAAAATAAAAGAAAAGGACAAGCTATATTCGCATCACACAGAAAAATGCCATAAAGAAGGTGAGAAGAGCAAAAATA
H4	97	3	63600958	AGACCGGCTGGCATTCTTCGGGACAGAGATATCTTTCAGATCCAATGGAGAAGCACTCCAGCAGCTATAATCAAGGTGGTTACAACCAAGACAGCTATGGATCAAGCAGTGGGTACAGTCAGCAGGCGAGTTTGGGGCCAACCTCAGATG
	65	7	54617638	CTTCAGGAACACAAGGCCCAGGCCTATCTGAAAGTCAATGCCAACAGCCATGCCCGCAGAATGCAGGCCTTCGAAGCCTCGCCCATGTGGCTGCAGGATATGAAGCCCCGCAAGAACGTCTCCGCAGCCATCCCCAGCAGCATCCATGGC
	65	12	113629469	CACAAATGGGCCTCAGGATCTCAGGCCTTCCACGTCAGGGGTGACCTTCCGGAGCCCCCTGGTGACTTCCAGGGCTCGCTCAGTTAGCATTTCAGTGCCATCTACCCCACGACGAGGTGGGGCCACCCAGAAACCAAAGCCCCCTTGGAA
	161	14	70381786	TGTTGGCCAGGCTGGTCTCCAACTCCTGACCTCAGGTGATCCACCCGCTTCAGCCTCCCAAAGTGCTGGGATTGCAGGCTTGAGTCACCGCGCCCGGCCAGTTTACACATTCTGTCCTCAAACTTCCAACACATTGGTCCTCCTTCTTCA
	161	16	25182094	GTACAACAGGTTACCTCGAGCTGAAGTGAGCAGGTATGGGGTTGGTGAGCGTCAGCTTGATGGGCATTCATTGTGAACTCAAGGCATGATTGCCAGTATTCTTTCATGCAGAAAAACAGAACAATATGTGGAGATTTCATTATGCCTTTG
	161	20	23213732	CCCTCTCCACGTCCCCGTGGCACTTTGCTCATGAGGCTATCAGAGAATGTCTCAGACCACGTGGATCATGTTGGGGCCCAACCCCAGCTGTGACTGCTGTCCAGGCCTTTGTGTAGGAAGTGAGGGCAGGCTGGGGCAGGGCTTTGCGGG
C5	129	15	49833908	TCACCAGCACCATGCCTAAATCTTCTTTAAATTCATCATTAAAAAGGTAACTCGATTCTGGAAATGCTTCATAGAACGTTGCATATATGGCTTGTGCCAAACAATCAGCATATATCTACCAAAATAAAATCAAATTAGATTAATTTTATA
H5	161	3	181874865	CAATTGCAAATGCAGCAATCACCTACCTTCTGCCTCGATCTTGCTGGGAACTACAGACTGGCACTCTTCCTATTCAGCTATCTTGCCAGCCACCTATTTATTTATTTTTATTCTTTTCTCCTTTTGAGGAGGCATAATAATTACTTCAA
	97	14	36622991	GCCAGACATGGTGCCTCACCTGTAATTCCAGTGCCTTGGGAGGCCAAGGTGGGAAGATTGTTCAAGCCCTGGAGTTTGAGAACAGCTTGGGCAACGTGTCGAGACTCCATCTCTCCAAAAAAATACAAAAATTAGCCAGTTGTGATGGTG
	129	15	49833908	GTACTAGGAGGCTGTAGGCATAAATTGGTCTGTTCACCAGCACCATGCCTAAATCTTCTTTAAATTCATCATTAAAAAGGTAACTCGATTCTGGAAATGCTTCATAGAACGTTGCATATATGGCTTGTGCCAAACAATCAGCATATATCT
	129	15	49833912	TGTAGGCATAAATTGGTCTGTGCACCAGCACCATGCCTAAATCGTCTTTAAAGTCATCATTAAAAAGGTAACTCGAGTCTGGCAATGCTTCATAGAACGTTGCATATATGGCTTGTGCCAAACAATCAGCATATATCTACCAAAATAAAA
	65	15	49833933	AGGTAACTCGATTCTGGAAATGCTTCATAGAACGTTGCATATATGGCTTGTGCCAAACAATCAGCATATATCTACCAAAATAAAATCAAATTAGATTAATTTTATATTTTTAAATTTTAATATATGTCAGCTGCCAAAGAAATTATTATT
	129	15	49833992	ATCAGCATATATCTACCAAAATAAAAAAGAATGAGATTAATCTTATATTTTTAAATATTAATCAATATCACCTGCAAAAGAAATTATTATACTTTAAATATGCCTTGCAATTTATTAACCGAATAAATATTCGCCAGTTTTTAAATCGTA
H6	129	5	17704483	AACAAACCCTATTTTTGTAAGGGAAAAACACTTTTCAAATTGCATGGTGCTGGTGAACACACCAATTTATGCCTACAGCCTCGTAATGATTAACTACATACAAAGACCTTTAACCTAACCTCCTCCCCCAACTCCTCCCACTCATTAAAC

Supplementary Table 4: Distribution of driver mutations of 7 H-ChC patients

Patient	Hugo_Symbol	Chromosome	Position	Variant_Classification	Variant_Type	Gene_Description
P1	PTPRC	1	198718543	Splice_Site	SNP	protein tyrosine phosphatase, receptor type, C
	LRIF1	1	111494203	Missense_Mutation	SNP	ligand dependent nuclear receptor interacting factor 1
	EML4	2	42553333	Missense_Mutation	SNP	echinoderm microtubule associated protein like 4
	AKAP9	7	91708531	Nonsense_Mutation	SNP	A kinase (PRKA) anchor protein 9
	FGFR1	8	38297829	Missense_Mutation	SNP	fibroblast growth factor receptor 1
	USP28	11	113702667	Missense_Mutation	SNP	ubiquitin specific peptidase 28
	MEF2A	15	100214595	Splice_Site	SNP	myocyte enhancer factor 2A
	GRIN2A	16	9934887	Missense_Mutation	SNP	glutamate receptor, ionotropic, N-methyl D-aspartate 2A

	CDH1	16	68867343	Missense_Mutation	SNP	cadherin 1, type 1, E-cadherin (epithelial)
	TP53	17	7579466	Frame_Shift_Del	DEL	tumor protein p53
	ARHGAP35	19	47424519	Missense_Mutation	SNP	Rho GTPase activating protein 35
	NF2	22	30000099	Nonsense_Mutation	SNP	neurofibromin 2 (merlin)
	HUWE1	X	53622340	Missense_Mutation	SNP	HECT, UBA and WWE domain containing 1, E3 ubiquitin protein ligase
P2	H3F3A	1	226259113	Missense_Mutation	SNP	H3 histone, family 3A
	CBLB	3	105459347	Missense_Mutation	SNP	Cbl proto-oncogene B, E3 ubiquitin protein ligase
	PTPN13	4	87701584	Missense_Mutation	SNP	protein tyrosine phosphatase, non-receptor type 13 (APO-1/CD95 (Fas)-associated phosphatase)
	CARD11	7	2962791	Missense_Mutation	SNP	caspase recruitment domain family, member 11
	SND1	7	127714722	Missense_Mutation	SNP	staphylococcal nuclease and tudor domain containing 1
	TCEA1	8	54900719	Missense_Mutation	SNP	transcription elongation factor A (SII), 1
	SPTAN1	9	131339476	Missense_Mutation	SNP	spectrin, alpha, non-erythrocytic 1
	ZFHX3	16	72992087	Missense_Mutation	SNP	zinc finger homeobox 3
	OLIG2	21	34400039	Missense_Mutation	SNP	oligodendrocyte lineage transcription factor 2
	DMD	X	32383144	Missense_Mutation	SNP	dystrophin
P3	SOS1	2	39234285	Nonsense_Mutation	SNP	son of sevenless homolog 1 (Drosophila)
	ZFP36L2	2	43452447	Missense_Mutation	SNP	ZFP36 ring finger protein-like 2
	ACKR3	2	237489593	Missense_Mutation	SNP	atypical chemokine receptor 3
	ACVR2A	2	148680663	Missense_Mutation	SNP	activin A receptor, type IIA
	FOXP1	3	71090546	Missense_Mutation	SNP	forkhead box P1
	CACNA1D	3	53844020	Missense_Mutation	SNP	calcium channel, voltage-dependent, L type, alpha 1D subunit
	PIK3CB	3	138461563	Missense_Mutation	SNP	phosphatidylinositol-4,5-bisphosphate 3-kinase, catalytic subunit beta
	PDGFRA	4	55151580	Missense_Mutation	SNP	platelet-derived growth factor receptor, alpha polypeptide
	METAP1	4	99955468	Missense_Mutation	SNP	methionyl aminopeptidase 1
	HOOK3	8	42828543	Splice_Site	SNP	hook microtubule-tethering protein 3
	ERCC4	16	14031664	Missense_Mutation	SNP	excision repair cross-complementation group 4
	TP53	17	7578492	Frame_Shift_Del	DEL	tumor protein p53
	PRPF8	17	1580871	Missense_Mutation	SNP	pre-mRNA processing factor 8
P4	RBM15	1	110883694	Missense_Mutation	SNP	RNA binding motif protein 15
	AFF3	2	100623895	Missense_Mutation	SNP	AF4/FMR2 family, member 3
	NEB	2	152471104	Splice_Site	SNP	nebulin
	QKI	6	163987774	Missense_Mutation	SNP	QKI, KH domain containing, RNA binding
	SYNE1	6	152576061	Missense_Mutation	SNP	spectrin repeat containing, nuclear envelope 1
	FANCC	9	97876991	Frame_Shift_Del	DEL	Fanconi anemia, complementation group C
	KAT6B	10	76602916	Missense_Mutation	SNP	K(lysine) acetyltransferase 6B
	MYO5A	15	52675324	Missense_Mutation	SNP	myosin VA (heavy chain 12, myoxin)
	TP53	17	7577534	Missense_Mutation	SNP	tumor protein p53
	MED13	17	60060470	Missense_Mutation	SNP	mediator complex subunit 13
	RBM10	X	47039693	Missense_Mutation	SNP	RNA binding motif protein 10
P5	MTOR	1	11307750	Missense_Mutation	SNP	mechanistic target of rapamycin (serine/threonine kinase)
	ACVR2A	2	148677851	Missense_Mutation	SNP	activin A receptor, type IIA
	KALRN	3	124153349	Missense_Mutation	SNP	kalirin, RhoGEF kinase

	SYNE1	6	152555004	Missense_Mutation	SNP	spectrin repeat containing, nuclear envelope 1
	RUNX1T1	8	93029564	Missense_Mutation	SNP	runt-related transcription factor 1; translocated to, 1 (cyclin D-related)
	JMJD1C	10	65024454	Missense_Mutation	SNP	jumonji domain containing 1C
	CIITA	16	11016346	Missense_Mutation	SNP	class II, major histocompatibility complex, transactivator
	MED13	17	60060470	Missense_Mutation	SNP	mediator complex subunit 13
	NFATC2	20	50140613	Missense_Mutation	SNP	nuclear factor of activated T-cells, cytoplasmic, calcineurin-dependent 2
	5-Sep	22	19707175	Missense_Mutation	SNP	septin 5
P6	ASPM	1	197073312	Missense_Mutation	SNP	asp (abnormal spindle) homolog, microcephaly associated (Drosophila)
	LMNA	1	156106013	Missense_Mutation	SNP	lamin A/C
	AHDC1	1	27874065	Missense_Mutation	SNP	AT hook, DNA binding motif, containing 1
	FBXO11	2	48060168	Missense_Mutation	SNP	F-box protein 11
	GMPS	3	155654183	Missense_Mutation	SNP	guanine monphosphate synthase
	PTPN13	4	87637723	Missense_Mutation	SNP	protein tyrosine phosphatase, non-receptor type 13 (APO-1/CD95 (Fas)-associated phosphatase)
	PIK3R1	5	67589627	Missense_Mutation	SNP	phosphoinositide-3-kinase, regulatory subunit 1 (alpha)
	APC	5	112173481	Missense_Mutation	SNP	adenomatous polyposis coli
	NIPBL	5	37064849	Missense_Mutation	SNP	Nipped-B homolog (Drosophila)
	ARID1B	6	157527922	Missense_Mutation	SNP	AT rich interactive domain 1B (SWI1-like)
	MET	7	116340324	Missense_Mutation	SNP	MET proto-oncogene, receptor tyrosine kinase
	ADCY1	7	45697441	Missense_Mutation	SNP	adenylate cyclase 1 (brain)
	NCOA4	10	51581274	Missense_Mutation	SNP	nuclear receptor coactivator 4
	TP53BP1	15	43749192	Frame_Shift_Del	DEL	tumor protein p53 binding protein 1
	ANKRD11	16	89350273	Missense_Mutation	SNP	ankyrin repeat domain 11
	TP53	17	7577534	Missense_Mutation	SNP	tumor protein p53
	HUWE1	X	53607865	Missense_Mutation	SNP	HECT, UBA and WWE domain containing 1, E3 ubiquitin protein ligase
P7	ACSL6	5	131309062	Missense_Mutation	SNP	acyl-CoA synthetase long-chain family member 6
	CHN1	2	175664864	Missense_Mutation	SNP	chimerin 1
	BAP1	3	52440925	Splice_Site	SNP	BRCA1 associated protein-1 (ubiquitin carboxy-terminal hydrolase)
	CDKN2A	9	21971153	Nonsense_Mutation	SNP	cyclin-dependent kinase inhibitor 2A
	H3F3A	1	226259113	Missense_Mutation	SNP	H3 histone, family 3A

Supplementary Table 5: Information of significantly mutated genes for all 21 samples

Gene	Indels	SNVs	Tot Muts	Sample Affect	Sample Percent(%)	P-value CT	FDR CT
TP53	4	4	8	8	57.14	1.89E-18	3.63E-14
MUC12	0	8	8	4	28.57	2.30E-08	2.20E-04
BAI3	0	5	5	3	21.43	1.95E-07	0.001
CACNG4	0	4	4	4	28.57	4.93E-07	0.002
IFT122	0	4	4	2	14.29	5.23E-07	0.002
DAPK3	0	4	4	2	14.29	6.09E-07	0.002
SLITRK3	0	4	4	2	14.29	1.28E-06	0.003
ZNF804B	2	2	4	2	14.29	2.20E-06	0.005
MORC1	0	4	4	4	28.57	2.48E-06	0.005
SLC6A20	0	4	4	4	28.57	3.55E-06	0.007

BBS12	2	1	3	3	21.43	3.95E-06	0.007
NFATC3	0	4	4	4	28.57	4.70E-06	0.008
UCN3	0	2	2	2	14.29	5.80E-06	0.008
GRM1	0	4	4	4	28.57	6.02E-06	0.008
PTRH1	0	2	2	2	14.29	7.84E-06	0.01
ZNF266	0	2	2	2	14.29	9.97E-06	0.011
HDDC2	0	2	2	2	14.29	1.00E-05	0.011
ZNF354C	0	2	2	2	14.29	1.11E-05	0.012
LYZ	0	2	2	2	14.29	1.29E-05	0.013
FCGBP	1	5	6	5	35.71	1.33E-05	0.013
HOXC5	2	0	2	2	14.29	1.41E-05	0.013
C1orf174	2	0	2	2	14.29	1.72E-05	0.013
SYNDIG1	0	2	2	2	14.29	1.75E-05	0.013
CAMK2N1	0	2	2	2	14.29	1.69E-05	0.013
ANO3	0	4	4	3	21.43	1.59E-05	0.013
MAGEB5	2	0	2	2	14.29	1.84E-05	0.014
FOCAD	0	4	4	4	28.57	1.95E-05	0.014
ITM2A	0	2	2	2	14.29	2.15E-05	0.014
OPN1LW	0	2	2	2	14.29	2.13E-05	0.014
TUBE1	0	3	3	3	21.43	2.26E-05	0.014
C9	0	2	2	2	14.29	2.79E-05	0.016
SERPINA10	0	2	2	2	14.29	2.84E-05	0.016
POLR2J3	0	3	3	2	14.29	2.74E-05	0.016
G6PC	2	0	2	2	14.29	2.96E-05	0.017
FREM1	0	4	4	4	28.57	3.57E-05	0.02
CEP95	0	3	3	2	14.29	3.74E-05	0.02
FAM81B	2	0	2	2	14.29	4.31E-05	0.022
TTC5	2	0	2	1	7.14	4.47E-05	0.023
ARID1B	0	4	4	4	28.57	5.43E-05	0.023
MBTPS2	0	2	2	2	14.29	5.40E-05	0.023
SPON2	2	0	2	2	14.29	5.00E-05	0.023
SPRR2F	0	2	2	2	14.29	5.02E-05	0.023
MORN2	0	2	2	2	14.29	5.38E-05	0.023
HAND1	0	2	2	2	14.29	5.21E-05	0.023
IGSF9B	1	2	3	3	21.43	4.72E-05	0.023
WDR43	0	3	3	3	21.43	4.95E-05	0.023
RETSAT	0	3	3	2	14.29	5.71E-05	0.023
KRT37	2	0	2	2	14.29	6.36E-05	0.024
CSN3	0	2	2	2	14.29	6.27E-05	0.024
LCE6A	0	2	2	2	14.29	6.33E-05	0.024
SPANXN1	0	2	2	2	14.29	6.71E-05	0.024
FIBP	0	2	2	2	14.29	6.52E-05	0.024
PNRC2	0	2	2	2	14.29	6.72E-05	0.024
FAM227B	0	3	3	3	21.43	6.69E-05	0.024
PTPN13	0	4	4	4	28.57	7.28E-05	0.025
CELF1	2	0	2	2	14.29	7.34E-05	0.025
SLCO1A2	0	2	2	2	14.29	7.57E-05	0.025

NKX2-4	0	2	2	2	14.29	7.90E-05	0.026
B3GNT7	0	2	2	2	14.29	8.30E-05	0.027
APOLD1	0	2	2	2	14.29	8.45E-05	0.027
KPTN	2	0	2	2	14.29	8.77E-05	0.027
FADS6	0	2	2	2	14.29	8.90E-05	0.027
YJEFN3	0	2	2	2	14.29	8.66E-05	0.027
KLRF1	0	2	2	2	14.29	9.06E-05	0.027
TSC22D4	0	2	2	2	14.29	9.86E-05	0.029
ZNF676	0	3	3	3	21.43	0.000102064	0.029
TBC1D9	0	3	3	3	21.43	0.000107379	0.03
TAF4B	0	2	2	2	14.29	0.000119167	0.03
TRABD2A	0	2	2	2	14.29	0.000114394	0.03
ITGB3BP	0	2	2	2	14.29	0.000116281	0.03
C2orf74	0	2	2	2	14.29	0.000114453	0.03
WNT16	0	2	2	2	14.29	0.00011815	0.03
ANKRD20A4	0	3	3	3	21.43	0.000117434	0.03
DYRK1A	0	2	2	2	14.29	0.000121245	0.03
DEFB118	0	2	2	2	14.29	0.000122618	0.03
BHLHE41	2	0	2	2	14.29	0.000127382	0.031
FANCC	2	0	2	2	14.29	0.000131668	0.031
LCN9	0	2	2	2	14.29	0.000130193	0.031
GNA13	0	2	2	2	14.29	0.000132979	0.031
EIF1AD	0	2	2	2	14.29	0.000139152	0.032
PIWIL1	0	2	2	2	14.29	0.000154611	0.034
ENTPD8	2	0	2	2	14.29	0.000153371	0.034
EIF3M	0	2	2	2	14.29	0.00015356	0.034
FOXD4	0	2	2	2	14.29	0.000156394	0.034
RBMS1	0	2	2	2	14.29	0.000156867	0.034
CNTNAP3B	0	3	3	2	14.29	0.000159983	0.034
DUSP10	0	2	2	2	14.29	0.000168457	0.036
CDC42EP2	0	2	2	2	14.29	0.000183254	0.037
FAM189A1	0	2	2	2	14.29	0.000180867	0.037
GNAT3	0	2	2	2	14.29	0.000183825	0.037
SSTR4	0	2	2	2	14.29	0.000182287	0.037
MYL2	0	2	2	2	14.29	0.000190567	0.038
ADAM30	2	0	2	2	14.29	0.000203413	0.039
KRTAP1-1	0	2	2	2	14.29	0.000205031	0.039
PPP2R5D	0	2	2	2	14.29	0.00020066	0.039
RTL1	1	2	3	3	21.43	0.000201809	0.039
ANKRD30A	0	3	3	3	21.43	0.000205647	0.039
CCDC157	2	0	2	2	14.29	0.000211009	0.039
SEBOX	0	2	2	2	14.29	0.000212213	0.039
PRAMEF4	0	2	2	2	14.29	0.000215178	0.039
EYS	0	4	4	4	28.57	0.000214498	0.039
CCDC53	0	2	2	2	14.29	0.000227426	0.04
ZNF25	0	2	2	2	14.29	0.000226891	0.04
CCDC105	0	2	2	2	14.29	0.000236955	0.041

PRH2	0	2	2	2	14.29	0.000235786	0.041
BPHL	0	2	2	2	14.29	0.000249462	0.043
PHKB	0	2	2	2	14.29	0.000269054	0.044
DCUN1D2	0	2	2	2	14.29	0.000270136	0.044
UCMA	0	2	2	2	14.29	0.000270618	0.044
THEG5	0	2	2	2	14.29	0.000261598	0.044
EMCN	0	2	2	2	14.29	0.000265665	0.044
TMEM150C	0	2	2	2	14.29	0.000279082	0.045
SPRED3	0	2	2	2	14.29	0.000284806	0.045
TMEM202	0	2	2	2	14.29	0.000289391	0.045
ARHGAP15	0	2	2	2	14.29	0.000288743	0.045
NTF4	0	2	2	2	14.29	0.000298452	0.046
PSKH2	0	2	2	2	14.29	0.000294796	0.046
SIRPD	0	2	2	2	14.29	0.000297286	0.046
SPIN4	0	2	2	2	14.29	0.000301099	0.046
DCAF12L1	0	2	2	2	14.29	0.000302503	0.046
ASNA1	0	2	2	2	14.29	0.000307501	0.046
ASXL3	0	3	3	3	21.43	0.000307308	0.046
CHSY3	0	2	2	2	14.29	0.000311032	0.046
GCC1	0	2	2	2	14.29	0.000325769	0.048
ENTPD2	0	2	2	2	14.29	0.000330228	0.048
IZUMO1	0	2	2	2	14.29	0.000347035	0.05
SPATA17	0	2	2	2	14.29	0.000352117	0.05
ALOXE3	2	0	2	2	14.29	0.000360303	0.05
ACHE	0	2	2	2	14.29	0.000360459	0.05
TMPRSS11E	0	2	2	2	14.29	0.000364077	0.05
TSPAN17	0	2	2	2	14.29	0.000370005	0.05
CD40	0	2	2	2	14.29	0.000367982	0.05
EDIL3	0	2	2	2	14.29	0.000372184	0.05
TRIM10	0	2	2	2	14.29	0.000377832	0.051
TACSTD2	0	2	2	2	14.29	0.000382951	0.051
NR0B1	0	2	2	2	14.29	0.000388919	0.051
SLC13A4	0	2	2	2	14.29	0.000390587	0.051
FAT2	0	5	5	5	35.71	0.000402312	0.052
HOXB4	0	2	2	2	14.29	0.000410997	0.053
RAB3C	0	2	2	2	14.29	0.000416744	0.054
RRBP1	2	0	2	2	14.29	0.000434876	0.055
SUMF2	0	2	2	2	14.29	0.000438831	0.055
TMEM221	0	2	2	2	14.29	0.000440556	0.055
USP14	0	2	2	2	14.29	0.000435127	0.055
TOM1L1	0	2	2	2	14.29	0.000467924	0.058
FOXF2	0	2	2	2	14.29	0.000473232	0.059
CBX6	0	2	2	2	14.29	0.00047731	0.059
RASEF	0	2	2	2	14.29	0.000479411	0.059
LIME1	0	2	2	2	14.29	0.000484785	0.059
OLIG2	0	2	2	2	14.29	0.000486167	0.059
POMC	0	2	2	2	14.29	0.000498573	0.06

LRCH2	0	2	2	2	14.29	0.000501891	0.06
NPHS2	0	2	2	2	14.29	0.000514701	0.061
GKAP1	0	2	2	2	14.29	0.000518927	0.061
SLC35F4	0	2	2	2	14.29	0.000523344	0.061
ERCC6	2	0	2	2	14.29	0.000545376	0.062
KIAA0196	2	0	2	2	14.29	0.000547905	0.062
PATZ1	0	2	2	2	14.29	0.000550606	0.062
ANAPC7	0	2	2	2	14.29	0.000548609	0.062
SCAMP2	0	2	2	2	14.29	0.00057526	0.063
BTN2A2	0	2	2	2	14.29	0.000572275	0.063
KCTD9	0	2	2	2	14.29	0.000584582	0.063
ITFG1	0	2	2	2	14.29	0.000568781	0.063
GPR15	0	2	2	2	14.29	0.000568357	0.063
TRIM58	0	2	2	2	14.29	0.000579473	0.063
SGCZ	0	2	2	2	14.29	0.000581678	0.063
TDRD3	0	2	2	2	14.29	0.000567356	0.063
LPCAT1	0	2	2	2	14.29	0.000582255	0.063
PCDHGA7	0	2	2	2	14.29	0.00056207	0.063
JADE2	0	2	2	2	14.29	0.000588393	0.063
NLRX1	2	0	2	2	14.29	0.000591886	0.063
ITLN1	0	2	2	2	14.29	0.000595424	0.063
TMEM191C	0	2	2	2	14.29	0.000612229	0.064
LOXL1	0	2	2	2	14.29	0.000618492	0.064
NUP37	0	2	2	2	14.29	0.000622142	0.064
WRAP73	0	2	2	2	14.29	0.000620133	0.064
APLP1	0	2	2	2	14.29	0.000610795	0.064
ZSWIM2	0	2	2	2	14.29	0.000631775	0.064
TRIM43	0	2	2	2	14.29	0.000637855	0.065
SNX30	0	2	2	2	14.29	0.000659993	0.066
CYBA	0	2	2	2	14.29	0.000662101	0.066
SLFN12L	0	2	2	2	14.29	0.000659491	0.066
RHAG	0	2	2	2	14.29	0.000668387	0.066
KIAA1147	0	2	2	2	14.29	0.000683226	0.067
PRDX1	0	2	2	2	14.29	0.000680461	0.067
GNPTAB	2	0	2	2	14.29	0.000734518	0.068
BICD2	0	2	2	2	14.29	0.000712557	0.068
OTX2	0	2	2	2	14.29	0.00073621	0.068
NOTO	0	2	2	2	14.29	0.000693955	0.068
ASB11	0	2	2	2	14.29	0.000704046	0.068
CPA5	0	2	2	2	14.29	0.000707585	0.068
DPY19L2	0	2	2	2	14.29	0.000720027	0.068
PLBD1	0	2	2	2	14.29	0.000734382	0.068
POLR2B	0	2	2	2	14.29	0.000709032	0.068
PABPC4L	0	2	2	2	14.29	0.000735588	0.068
SRRM4	0	2	2	2	14.29	0.00072417	0.068
PLEKHH2	0	5	5	3	21.43	0.000715322	0.068
KRTAP10-2	0	2	2	2	14.29	0.000698752	0.068

ZNF468	1	1	2	2	14.29	0.000695283	0.068
ROR2	0	2	2	2	14.29	0.00075095	0.069
ACTB	0	2	2	2	14.29	0.000762798	0.069
MUC4	0	5	5	2	14.29	0.000761952	0.069
STX3	0	2	2	2	14.29	0.000769512	0.069
MREG	0	2	2	2	14.29	0.000776168	0.07
P2RX4	0	2	2	2	14.29	0.000800991	0.071
CSMD3	0	4	4	4	28.57	0.000800089	0.071
GSE1	2	0	2	2	14.29	0.000817261	0.072
SEC23A	0	2	2	2	14.29	0.000817582	0.072
TMEM8A	0	2	2	2	14.29	0.000811452	0.072
ATP1B2	0	2	2	2	14.29	0.000833975	0.073
SLC50A1	0	2	2	2	14.29	0.000836647	0.073
ZNF420	0	2	2	2	14.29	0.00084127	0.073
CDCA7	0	2	2	2	14.29	0.000859627	0.074
GALE	0	2	2	2	14.29	0.000875642	0.075
CR2	0	2	2	2	14.29	0.000890027	0.076
APOA5	0	2	2	2	14.29	0.000921471	0.078
PRKCZ	0	2	2	2	14.29	0.000957771	0.081
PCDHGA6	0	2	2	2	14.29	0.000954462	0.081
SOS1	0	2	2	2	14.29	0.00098324	0.082
IFIT2	0	2	2	2	14.29	0.000989334	0.082
VRTN	0	2	2	2	14.29	0.000990352	0.082
VCAN	0	3	3	3	21.43	0.00098821	0.082
OTOL1	0	2	2	2	14.29	0.000995771	0.082
CDKN2A	0	2	2	2	14.29	0.001008447	0.083
GLCE	0	2	2	2	14.29	0.001042228	0.085
RTTN	0	3	3	3	21.43	0.001041115	0.085
ALPK1	0	2	2	2	14.29	0.001047775	0.085
ZIM2	0	2	2	2	14.29	0.001078656	0.087
MEGF9	0	2	2	2	14.29	0.001102219	0.088
ZNF326	0	2	2	2	14.29	0.001106143	0.088
TLK1	0	2	2	2	14.29	0.00112286	0.089
ADSS	0	2	2	2	14.29	0.001124834	0.089
SLC18B1	0	2	2	2	14.29	0.001127092	0.089
WDR92	0	2	2	2	14.29	0.001154585	0.09
SCNN1G	0	2	2	2	14.29	0.001156475	0.09
MST1L	0	2	2	2	14.29	0.00114868	0.09
TEF	0	2	2	2	14.29	0.001190603	0.092
ZNF79	0	2	2	2	14.29	0.001194198	0.092
KLHL10	0	2	2	2	14.29	0.001195193	0.092
VEPH1	0	2	2	2	14.29	0.001210041	0.092
ZFP30	0	2	2	2	14.29	0.001213081	0.092
ACVR2A	0	2	2	2	14.29	0.001206077	0.092
ITGA3	0	2	2	2	14.29	0.001238235	0.093
WDR44	0	2	2	2	14.29	0.001237092	0.093
BRINP2	0	2	2	2	14.29	0.001244125	0.093

C8orf34	0	2	2	2	14.29	0.001268928	0.095
CAMSAP3	0	2	2	2	14.29	0.001307677	0.096
FAM160A1	0	2	2	2	14.29	0.001305693	0.096
DGAT2	0	2	2	2	14.29	0.001296696	0.096
EGFLAM	0	2	2	2	14.29	0.001294925	0.096
HRCT1	1	0	1	1	7.14	0.001305143	0.096
FBXO11	0	2	2	2	14.29	0.001324474	0.097
HDAC7	0	2	2	2	14.29	0.001345368	0.098
TENM1	0	3	3	3	21.43	0.001344464	0.098
MYO1A	0	2	2	2	14.29	0.001379831	0.099
TTC13	0	2	2	2	14.29	0.001376382	0.099
SPAG6	0	2	2	2	14.29	0.001377826	0.099
ZNF263	0	2	2	2	14.29	0.001377445	0.099
AMIGO2	0	2	2	2	14.29	0.001408198	0.1
RPAIN	0	2	2	2	14.29	0.001410384	0.1
DACT1	0	2	2	2	14.29	0.001428369	0.101
POF1B	0	2	2	2	14.29	0.001465931	0.103
KIF23	0	2	2	2	14.29	0.001475573	0.104
AQR	0	2	2	2	14.29	0.001493906	0.104
FRMD6	0	2	2	2	14.29	0.001491786	0.104
FANCL	0	2	2	2	14.29	0.001497522	0.104
CYP39A1	0	2	2	2	14.29	0.00150304	0.104
PPP1R16B	0	2	2	2	14.29	0.001600674	0.11
FAM73B	0	2	2	2	14.29	0.001632055	0.11
SLC7A7	0	2	2	2	14.29	0.001642959	0.11
BAP1	0	2	2	2	14.29	0.001616456	0.11
HSPA5	0	2	2	2	14.29	0.001631156	0.11
POFUT2	0	2	2	2	14.29	0.001603019	0.11
SOBP	0	2	2	2	14.29	0.001640763	0.11
SH2D7	0	2	2	2	14.29	0.001611836	0.11
SEC14L2	0	2	2	2	14.29	0.001634566	0.11
TP53BP1	2	0	2	2	14.29	0.00166515	0.111
ESPL1	0	3	3	3	21.43	0.001671114	0.111
FOXP4	0	2	2	2	14.29	0.001683886	0.112
RBM46	0	2	2	2	14.29	0.001691468	0.112
PDIA5	0	2	2	2	14.29	0.001757638	0.116
SLC19A3	0	2	2	2	14.29	0.00176972	0.116
XYLT1	0	2	2	2	14.29	0.001769511	0.116
ABCA13	0	4	4	4	28.57	0.001800661	0.117
SLC7A11	0	2	2	2	14.29	0.00182494	0.118
PCDHGA2	0	2	2	2	14.29	0.001826002	0.118
ITGA4	0	2	2	2	14.29	0.001836816	0.119
LRP2	0	4	4	2	14.29	0.001848666	0.119
DGCR8	0	2	2	2	14.29	0.00186471	0.12
ZNF573	0	2	2	2	14.29	0.001871943	0.12
SYT10	0	2	2	2	14.29	0.00188681	0.12
ATP13A3	0	2	2	2	14.29	0.001884501	0.12

PLA2G4E	0	2	2	2	14.29	0.001912645	0.121
RNF225	0	2	2	2	14.29	0.001912667	0.121
FZD9	0	2	2	2	14.29	0.001943085	0.121
EPHB3	0	2	2	2	14.29	0.00194074	0.121
BRD7	0	2	2	1	7.14	0.001942263	0.121
PTPRU	0	2	2	2	14.29	0.001989522	0.123
VWA3B	0	2	2	2	14.29	0.001986824	0.123
MAGEC3	0	2	2	2	14.29	0.002053418	0.127
LRRIQ1	0	2	2	2	14.29	0.002065262	0.127
RELN	0	3	3	3	21.43	0.00209337	0.129
SCMH1	0	2	2	2	14.29	0.002113936	0.129
F2	0	2	2	2	14.29	0.002149085	0.131
PRPF40B	0	2	2	2	14.29	0.002187523	0.133
SLK	0	2	2	2	14.29	0.002198905	0.133
TGFBRAP1	0	2	2	2	14.29	0.002221018	0.134
ZXDB	0	2	2	2	14.29	0.002286671	0.138
RELA	0	2	2	2	14.29	0.002301261	0.138
SCN8A	2	0	2	2	14.29	0.002349316	0.139
DDX43	0	2	2	2	14.29	0.002336293	0.139
ANKS3	0	2	2	2	14.29	0.002345418	0.139
PARN	0	2	2	2	14.29	0.002349705	0.139
FYB	0	2	2	2	14.29	0.002345363	0.139
ANO4	0	2	2	2	14.29	0.002321688	0.139
EYA4	0	2	2	2	14.29	0.002400124	0.141
PIK3R1	0	2	2	2	14.29	0.002415518	0.142
UNC45B	0	2	2	2	14.29	0.002478806	0.145
RASGRP1	0	2	2	2	14.29	0.00250559	0.146
ABCA8	0	2	2	2	14.29	0.002540427	0.148
CFAP61	0	2	2	2	14.29	0.002577226	0.149
LRRC10B	0	2	2	2	14.29	0.002577874	0.149
METTL4	0	1	1	1	7.14	0.002622853	0.151
AASS	0	2	2	2	14.29	0.002666604	0.153
SDAD1	0	2	2	2	14.29	0.002681181	0.153
PCDHB13	0	2	2	2	14.29	0.002690603	0.153
RGS9	0	2	2	2	14.29	0.002695981	0.153
DMGDH	0	2	2	2	14.29	0.0027041	0.153
SPTAN1	0	2	2	2	14.29	0.002769202	0.157
INTS10	0	2	2	2	14.29	0.002799727	0.158
CAPN9	0	2	2	2	14.29	0.002833958	0.159
ZNF714	0	2	2	2	14.29	0.002840325	0.159
FAM21C	0	2	2	2	14.29	0.002880708	0.161
ADAMTS14	0	2	2	2	14.29	0.002910148	0.162
ITGB8	0	2	2	2	14.29	0.002924766	0.162
OPA1	0	2	2	2	14.29	0.002938211	0.163
KIAA0319	0	2	2	2	14.29	0.002966901	0.164
TAS1R2	0	2	2	2	14.29	0.003030844	0.167
MICALL2	0	2	2	2	14.29	0.003219064	0.177

SEC61G	0	1	1	1	7.14	0.003235677	0.177
ARMC5	0	2	2	2	14.29	0.003273929	0.179
PPP1R35	1	0	1	1	7.14	0.003320149	0.181
SLC24A3	0	2	2	2	14.29	0.003335032	0.181
MUC2	0	3	3	3	21.43	0.003351305	0.181
GAK	0	2	2	2	14.29	0.003406865	0.184
CARD11	0	2	2	2	14.29	0.003480545	0.187
HGFAC	0	2	2	2	14.29	0.003517695	0.189
IL17RE	0	2	2	2	14.29	0.00355156	0.189
DPP4	0	2	2	2	14.29	0.003567862	0.189
CBLB	0	2	2	2	14.29	0.003535447	0.189
KLHL13	0	2	2	2	14.29	0.003560664	0.189
C9orf84	0	2	2	2	14.29	0.003545911	0.189
NFYB	0	1	1	1	7.14	0.003588656	0.189
ANKS6	0	2	2	2	14.29	0.00366052	0.192
FBLN2	0	2	2	2	14.29	0.003661616	0.192
CPT1C	0	2	2	2	14.29	0.003680029	0.193
VPS41	0	2	2	2	14.29	0.003729687	0.195
CTIF	0	2	2	2	14.29	0.003741908	0.195
SYPL2	1	0	1	1	7.14	0.003819472	0.198

Supplementary Table 6: Distribution of predisposing genes for 7 H-ChC patients

Patient	Hugo_Symbol	Chromosome	Position	Ref_allele	Alt_allele	Variant_Classification	Variant_Type
P1	PTPRC	1	198711122	A	G	Missense_Mutation	SNP
	PDE4DIP	1	145075683	C	T	Nonsense_Mutation	SNP
	NOTCH2	1	120512245	C	T	Missense_Mutation	SNP
	AFF3	2	100210334	CGGCTGAGG	CGG	In_Frame_Del	DEL
	PBRM1	3	52584431	TCT	TCTCCT	Splice_Site	INS
	KDR	4	55972974	T	A	Missense_Mutation	SNP
	FGFR3	4	1803361	G	T	Missense_Mutation	SNP
	RANBP17	5	170336704	G	A	Missense_Mutation	SNP
	PDGFRB	5	149497177	T	C	Splice_Site	SNP
	FGFR4	5	176520243	G	A	Missense_Mutation	SNP
	MAP3K1	5	56177848	TCAACAACAACAACAACAACAACAACA	TCAACAACAACAACAACAACAACA	In_Frame_Del	DEL
	CCND3	6	41903782	A	C	Missense_Mutation	SNP
	DAXX	6	33287880	GCCTCCTCCTC	GCCTCCTC	In_Frame_Del	DEL
	CREB3L2	7	137612913	CTGGTGGT	CTGGT	In_Frame_Del	DEL
	AKAP9	7	91652178	AAAC	AAACAAC	In_Frame_Ins	INS
	POT1	7	124475296	TAAACAAAACAAAACAAAACAAAACAAAACAAAA	TAAACAAAACAAAACAAAACAAAACAAAA,TAAACAAAACAAAACAAAACAAAACAAAACAAAACAAAACAAA	Splice_Site	INS
	RECQL4	8	145737816	C	T	Missense_Mutation	SNP
	PTCH1	9	98209594	G	A	Missense_Mutation	SNP
	NOTCH1	9	139391403	C	T	Missense_Mutation	SNP

	CCDC6	10	61552692	G	T	Missense_Mutation	SNP
	CREB3L1	11	46342259	AGG	AGGG	Frame_Shift_Ins	INS
	MAML2	11	95825374	TTGCTGCTGCTGCTGCTGCTGCTGCTGCTGCTGCTGCTGCTG	TTGCTGCTGCTGCTGCTGCTGCTGCTGCTGCTG	In_Frame_Del	DEL
	ZNF384	12	6777069	TTGCTGCTGCTGCTGCTGCTGCTGCTGCTGCTGCTGCTGCTGCTGCTG	TTGCTGCTGCTGCTGCTGCTGCTGCTGCTGCTGCTGCTGCTG	In_Frame_Del	DEL
	CIITA	16	10989219	C	G	Missense_Mutation	SNP
	SETBP1	18	42456670	C	CTCTT	Frame_Shift_Ins	INS
	ASXL1	20	31019136	G	A	Missense_Mutation	SNP
	TMPRSS2	21	42852497	C	T	Missense_Mutation	SNP
	MN1	22	28194933	TTGCTGCTGCTGCTGCTGCTGCTGCTGCTG	TTGCTGCTGCTGCTGCTGCTGCTGCTGCTGCTG	In_Frame_Ins	INS
	MN1	22	28194894	CTGCTGTTGCTGTTGCTGTTGCTG	CTGCTGTTGCTGTTGCTG	In_Frame_Del	DEL
	CDC42EP1	22	37964408	CCAGCGCCTGCTGCAAACCCCTCAGC	CCAGC	In_Frame_Del	DEL
	CLTCL1	22	19189003	AC	ACC	Splice_Site	INS
P2	NTRK1	1	156814027	G	A	Missense_Mutation	SNP
	ALK	2	29917793	C	T	Missense_Mutation	SNP
	PBRM1	3	52584431	TCT	TCTCCT	Splice_Site	INS
	WWTR1	3	149238595	C	CTTAA	Nonsense_Mutation	INS
	SLC34A2	4	25675989	G	A	Missense_Mutation	SNP
	KDR	4	55972974	T	A	Missense_Mutation	SNP
	KDR	4	55979558	C	T	Missense_Mutation	SNP
	FGFR4	5	176520243	G	A	Missense_Mutation	SNP
	MAP3K1	5	56177848	TCAACAACAACAACAACAACAACAACA	TCAACAACAACAACAACAACAACA	In_Frame_Del	DEL
	ROS1	6	117650532	C	G	Missense_Mutation	SNP
	CCND3	6	41903782	A	C	Missense_Mutation	SNP
	CREB3L2	7	137612913	CTGGTGGT	CTGGT	In_Frame_Del	DEL
	POT1	7	124475296	TAAACAAAACAAAACAAAACAAAACAAAACAAAA	TAAACAAAACAAAACAAAACAAAACAAAACAAAACAAAA	Splice_Site	INS
	RECQL4	8	145737816	C	T	Missense_Mutation	SNP
	RECQL4	8	145738767	CGG	CG	Frame_Shift_Del	DEL
	PCM1	8	17827260	AC	ACC	Splice_Site	INS
	PTCH1	9	98209594	G	A	Missense_Mutation	SNP
	MLLT3	9	20414340	GCTACT	GCT	In_Frame_Del	DEL
	RET	10	43600607	C	A	Missense_Mutation	SNP
	KAT6B	10	76781905	GGAAGAAGAAGAAGAAGAAGAAGAA	GGAAGAAGAAGAAGAAGAAGAA	In_Frame_Del	DEL
	MAML2	11	95825374	TTGCTGCTGCTGCTGCTGCTGCTGCTGCTGCTGCTGCTGCTG	TTGCTGCTGCTGCTGCTGCTGCTGCTGCTGCTG,TTGCTGCTGCTGCTGCTGCTGCTGCTGCTG	In_Frame_Del	INS
	CREB3L1	11	46342259	AGG	AGGG	Frame_Shift_Ins	INS

	ALDH2	12	112241766	G	A	Missense_Mutation	SNP
	ZNF384	12	6777069	TTGCTGCTGCTGCTGCTGCTGCTGCTGCTGCTGCTGCTGCTGCTG	TTGCTGCTGCTGCTGCTGCTGCTGCTGCTGCTGCTGCTG	In_Frame_Del	DEL
	GOLGA5	14	93276667	T	C	Missense_Mutation	SNP
	NTRK3	15	88576178	C	T	Missense_Mutation	SNP
	CIITA	16	10989219	C	G	Missense_Mutation	SNP
	BRIP1	17	59820495	T	C	Missense_Mutation	SNP
	SETBP1	18	42456670	C	CTCTT	Frame_Shift_Ins	INS
	MAP2K2	19	4099225	G	A	Missense_Mutation	SNP
	CLTCL1	22	19189003	AC	ACC	Splice_Site	INS
	GATA1	X	48652565	C	A	Missense_Mutation	SNP
P3	ETV5	3	185783742	G	A	Missense_Mutation	SNP
	WWTR1	3	149238595	C	CTTAA	Nonsense_Mutation	INS
	KDR	4	55979558	C	T	Missense_Mutation	SNP
	KDR	4	55972974	T	A	Missense_Mutation	SNP
	MAP3K1	5	56177848	TCAACAACAACAACAACAACAACAACA	TCAACAACAACAACAACAACAACA	In_Frame_Del	DEL
	CCND3	6	41903782	A	C	Missense_Mutation	SNP
	AKAP9	7	91714216	A	G	Missense_Mutation	SNP
	CREB3L2	7	137612913	CTGGTGGT	CTGGT	In_Frame_Del	DEL
	POT1	7	124475296	TAAACAAAACAAAACAAAACAAAACAAAACAAAA	TAAACAAAACAAAACAAAACAAAACAAAACAAAACAAAA	Splice_Site	INS
	AKAP9	7	91652178	AAAC	AAACAAC	In_Frame_Ins	INS
	PTCH1	9	98209594	G	A	Missense_Mutation	SNP
	CCDC6	10	61552692	G	T	Missense_Mutation	SNP
	KAT6B	10	76781905	GGAAGAAGAAGAAGAAGAAGAAGAA	GGAAGAAGAAGAAGAAGAAGAA	In_Frame_Del	DEL
	NUP98	11	3726498	G	A	Missense_Mutation	SNP
	KMT2A	11	118307445	G	A	Missense_Mutation	SNP
	ATM	11	108192078	C	T	Missense_Mutation	SNP
	CREB3L1	11	46342259	AGG	AGGG	Frame_Shift_Ins	INS
	MAML2	11	95825374	TTGCTGCTGCTGCTGCTGCTGCTGCTGCTGCTGCTGCTG	TTGCTGCTGCTGCTGCTGCTGCTGCTGCTG	In_Frame_Del	DEL
	KMT2D	12	49426753	AGTTGCTGTTGCTGTTGC	AGTTGCTGTTGC	In_Frame_Del	DEL
	ZNF384	12	6777069	TTGCTGCTGCTGCTGCTGCTGCTGCTGCTGCTGCTGCTGCTGCTG	TTGCTGCTGCTGCTGCTGCTGCTGCTGCTGCTGCTGCTG	In_Frame_Del	DEL
	SETBP1	18	42456670	CA	CTCTTA	Frame_Shift_Ins	INS
	CLTCL1	22	19189003	AC	ACC	Splice_Site	INS
P4	RANBP2	2	109388225	C	A	Missense_Mutation	SNP
	MECOM	3	168861569	C	T	Missense_Mutation	SNP
	PBRM1	3	52584431	TCT	TCTCCT	Splice_Site	INS
	KDR	4	55972974	T	A	Missense_Mutation	SNP
	KDR	4	55979558	C	T	Missense_Mutation	SNP

	PDGFRA	4	55151537	G	C	Splice_Site	SNP
	PWWP2A	5	159546170	CCGGCGGCGG CGG	CCGGCGGCGG CGGCGGCGG	In_Frame_Ins	INS
	CCND3	6	41903782	A	C	Missense_Mutation	SNP
	ELN	7	73466166	G	A	Missense_Mutation	SNP
	CREB3L2	7	137612913	CTGGTGGT	CTGGT	In_Frame_Del	DEL
	POT1	7	124475296	TAAACAAAAC AAAACAAAAC AAAACAAAAC AAAA	TAAACAAAAC AAAACAAAAC AAAACAAAA	Splice_Site	DEL
	RECQL4	8	145737816	C	T	Missense_Mutation	SNP
	PTCH1	9	98209594	G	A	Missense_Mutation	SNP
	KAT6B	10	76781905	GGAAGAAGAA GAAGAAGAAG AAGAA	GGAAGAAGAA GAAGAAGAAG AA	In_Frame_Del	DEL
	MAML2	11	95825374	TTGCTGCTGCT GCTGCTGCTGC TGCTGCTGCTG CTGCTGCTG	TTGCTGCTGCT GCTGCTGCTGC TGCTGCTGCTG	In_Frame_Del	DEL
	CREB3L1	11	46342259	AGG	AGGG	Frame_Shift_Ins	INS
	KMT2D	12	49434910	A	T	Missense_Mutation	SNP
	ZNF384	12	6777069	TTGCTGCTGCT GCTGCTGCTGC TGCTGCTGCTG CTGCTGCTGCT G	TTGCTGCTGCT GCTGCTGCTGC TGCTGCTGCTG CTGCTGCTG	In_Frame_Del	DEL
	GPHN	14	67576863	C	G	Missense_Mutation	SNP
	TSC2	16	2134572	C	G	Missense_Mutation	SNP
	CIITA	16	10989219	C	G	Missense_Mutation	SNP
	RNF43	17	56436109	C	T	Missense_Mutation	SNP
	SETBP1	18	42456670	C	CTCTT	Frame_Shift_Ins	INS
	PPP2R1A	19	52725428	C	T	Missense_Mutation	SNP
	TMPRSS2	21	42852497	C	T	Missense_Mutation	SNP
	BCR	22	23603140	G	C	Missense_Mutation	SNP
	CLTCL1	22	19189003	AC	ACC	Splice_Site	INS
P5	NTRK1	1	156814027	G	A	Missense_Mutation	SNP
	HOXD11	2	176972817	G	A	Missense_Mutation	SNP
	FBXO11	2	48066912	T	C	Splice_Site	SNP
	MITF	3	69788786	T	C	Missense_Mutation	SNP
	WWTR1	3	149238595	C	CTTAA	Nonsense_Mutation	INS
	KDR	4	55972974	T	A	Missense_Mutation	SNP
	PHOX2B	4	41747995	CGCCGCTGCC GCTGCCGCCG CCGCCGCTGC CGC	CGCCGC	In_Frame_Del	DEL
	FGFR4	5	176520243	G	A	Missense_Mutation	SNP
	IL7R	5	35867500	G	A	Missense_Mutation	SNP
	MAP3K1	5	56177848	TCAACAACAA CAACAACAAC AACAACA	TCAACAACAA CAACAACAAC AACA	In_Frame_Del	DEL
	ROS1	6	117650532	C	G	Missense_Mutation	SNP
	CCND3	6	41903782	A	C	Missense_Mutation	SNP
	CREB3L2	7	137612913	CTGGTGGT	CTGGT	In_Frame_Del	DEL
	POT1	7	124475296	TAAACAAAAC AAAACAAAAC AAAACAAAAC AAAA	TAAACAAAAC AAAACAAAAC AAAACAAAAC AAAACAAAA	Splice_Site	INS

	RECQL4	8	145738767	CGG	CG	Frame_Shift_Del	DEL
	PTCH1	9	98209594	G	A	Missense_Mutation	SNP
	CCDC6	10	61552692	G	T	Missense_Mutation	SNP
	RET	10	43600607	C	A	Missense_Mutation	SNP
	KAT6B	10	76781905	GGAAGAAGAAGAAGAAGAAGAAGAA	GGAAGAAGAAGAAGAAGAAGAA	In_Frame_Del	DEL
	EXT2	11	44129444	G	A	Missense_Mutation	SNP
	CREB3L1	11	46342259	AGG	AGGG	Frame_Shift_Ins	INS
	MAML2	11	95825374	TTGCTGCTGCTGCTGCTGCTGCTGCTGCTGCTGCTGCTGCTG	TTGCTGCTGCTGCTGCTGCTGCTGCTGCTGCTG	In_Frame_Del	DEL
	COL2A1	12	48368604	C	T	Missense_Mutation	SNP
	ZNF384	12	6777069	TTGCTGCTGCTGCTGCTGCTGCTGCTGCTGCTGCTGCTGCTGCTG	TTGCTGCTGCTGCTGCTGCTGCTGCTGCTGCTGCTGCTGCTG,TTGCTGCTGCTGCTGCTGCTGCTGCTGCTGCTGCTGCTG	In_Frame_Del	INS
	TRIP11	14	92460227	C	T	Missense_Mutation	SNP
	CIITA	16	10989219	C	G	Missense_Mutation	SNP
	CIITA	16	11001421	C	A	Missense_Mutation	SNP
	TSC2	16	2115529	C	T	Missense_Mutation	SNP
	FANCA	16	89836354	G	A	Missense_Mutation	SNP
	TMPRSS2	21	42852497	C	T	Missense_Mutation	SNP
	CLTCL1	22	19189003	AC	ACC	Splice_Site	INS
P6	SH3GL1	19	4363448	G	A	Missense_Mutation	SNP
	NOTCH1	9	139399458	G	A	Missense_Mutation	SNP
	NTRK1	1	156844186	C	A	Missense_Mutation	SNP
	FANCA	16	89849320	T	C	Missense_Mutation	SNP
	PTCH1	9	98209594	G	A	Missense_Mutation	SNP
	CCND3	6	41903782	A	C	Missense_Mutation	SNP
	BCR	22	23610679	G	A	Missense_Mutation	SNP
	AXIN2	17	63531768	G	A	Missense_Mutation	SNP
	RECQL4	8	145737816	C	T	Missense_Mutation	SNP
	GRIN2A	16	9857974	C	T	Missense_Mutation	SNP
	TMPRSS2	21	42852497	C	T	Missense_Mutation	SNP
	CIITA	16	11001757	C	T	Missense_Mutation	SNP
	PDE4DIP	1	145075683	C	T	Nonsense_Mutation	SNP
	CIITA	16	10989219	C	G	Missense_Mutation	SNP
	CCDC6	10	61552692	G	T	Missense_Mutation	SNP
	WHSC1	4	1920323	G	T	Missense_Mutation	SNP
	HOXA11	7	27224469	C	T	Missense_Mutation	SNP
	RECQL4	8	145738767	CGG	CG	Frame_Shift_Del	DEL
	PHOX2B	4	41747989	AGCTGCCGCCGCTGCCGCTGCCGCCGCCGCCGCTGCCGC	AGCTGCCGCCGCCGCCGCTGCCGC	In_Frame_Ins	DEL
	TAF15	17	34171662	TGGCTATGGTGGGGACAGAGGCGGCGGCTATGGTGGGGACAGAGGAGGCGGCTATGG	TGGCTATGGTGGGGACAGAGGAGGCGGCTATGG	In_Frame_Ins	DEL

	POT1	7	124475296	TAAACAAAAC AAAACAAAAC AAAACAAAAC AAAA	TAAACAAAAC AAAACAAAAC AAAACAAAAC AAAACAAAA	Splice_Site	INS
	CDC42EP1	22	37964408	CCAGCGCCTG CTGCAAACCC CTCAGC	CCAGC	In_Frame_Del	DEL
	AKAP9	7	91652178	AAAC	AAACAAC	In_Frame_Ins	INS
	MAML2	11	95825374	TTGCTGCTGCT GCTGCTGCTGC TGCTGCTGCTG CTGCTGCTG	TTGCTGCTGCT GCTGCTGCTGC TGCTGCTGCTG	In_Frame_Del	DEL
	FUS	16	31196402	TGGCGGCGGC GGCGGCGGCG G	TGGCGGCGGC GGCGGCGGCG GCGG	In_Frame_Ins	INS
	ZNF384	12	6777069	TTGCTGCTGCT GCTGCTGCTGC TGCTGCTGCTG CTGCTGCTGCT G	TTGCTGCTGCT GCTGCTGCTGC TGCTGCTGCTG CTGCTGCTG	In_Frame_Del	DEL
	CREB3L1	11	46342259	AGG	AGGG	Frame_Shift_Ins	INS
	KAT6B	10	76781905	GGAAGAAGAA GAAGAAGAAG AAGAA	GGAAGAAGAA GAAGAAGAAG AA	In_Frame_Del	DEL
	CLTCL1	22	19189003	AC	ACC	Splice_Site	INS
	PCM1	8	17827260	AC	ACC	Splice_Site	INS
	SETBP1	18	42456670	C	CTCTT	Frame_Shift_Ins	INS
	CREB3L2	7	137612913	CTGGTGGT	CTGGT	In_Frame_Del	DEL
P7	ATIC	2	216191608	A	G	Missense_Mutation	SNP
	NCOA1	2	24952478	TCTCCT	TCT	In_Frame_Del	DEL
	PBRM1	3	52584431	TCT	TCTCCT	Splice_Site	INS
	KDR	4	55972974	T	A	Missense_Mutation	SNP
	PHOX2B	4	41747989	AGCTGCCGCC GCTGCCGCTG CCGCCGCCGC CGCTGCCGC	AGCTGCCGCC GCCGCCGCTG CCGC	In_Frame_Ins	DEL
	CCND3	6	41903782	A	C	Missense_Mutation	SNP
	TFEB	6	41652540	G	T	Missense_Mutation	SNP
	CREB3L2	7	137612913	CTGGTGGT	CTGGT	In_Frame_Del	DEL
	POT1	7	124475296	TAAACAAAAC AAAACAAAAC AAAACAAAAC AAAA	TAAACAAAAC AAAACAAAAC AAAACAAAA,T AAACAAAACA AAACAAAACA AAACAAAACA AAACAAAACA AAA	Splice_Site	INS
	TRRAP	7	98490062	G	A	Missense_Mutation	SNP
	IKBKB	8	42177163	G	A	Missense_Mutation	SNP
	PCM1	8	17827260	AC	ACC	Splice_Site	INS
	RECQL4	8	145738767	CGG	CG	Frame_Shift_Del	DEL
	CCDC6	10	61552692	G	T	Missense_Mutation	SNP
	CREB3L1	11	46342259	AGG	AGGG	Frame_Shift_Ins	INS
	DDX10	11	108788634	GTGATGATGA TGATGATGAT GATGATG	GTGATGATGA TGATGATGAT GATGATGATG ATG	In_Frame_Ins	INS
	MAML2	11	95825374	TTGCTGCTGCT GCTGCTGCTGC TGCTGCTGCTG CTGCTGCTG	TTGCTGCTGCT GCTGCTGCTGC TGCTGCTGCTG	In_Frame_Del	DEL
	KDM5A	12	432374	TACAAAAAAA AAAAAAAAAA	TAAAAAAAAA AAAAAA	Splice_Site	DEL

				AAAAAAAA			
	KDM5A	12	432374	TACAAAAAAAAAAAAAAAAAAAAAAAAAAA	TAAAAAAAAAAAAAAAAA	Splice_Site	DEL
	TBX3	12	115112055	G	A	Missense_Mutation	SNP
	ZNF384	12	6777069	TTGCTGCTGCTGCTGCTGCTGCTGCTGCTGCTGCTGCTGCTGCTGCTG	TTGCTGCTGCTGCTGCTGCTGCTGCTGCTGCTGCTGCTGCTG,TTGCTGCTGCTGCTGCTGCTGCTGCTGCTGCTGCTGCTGCTGCTGCTGCTGCTG	In_Frame_Del	INS
	NTRK3	15	88576178	C	T	Missense_Mutation	SNP
	CIITA	16	10989219	C	G	Missense_Mutation	SNP
	SETBP1	18	42456670	C	CTCTT	Frame_Shift_Ins	INS
	CLTCL1	22	19189003	AC	ACC	Splice_Site	INS
	MN1	22	28194933	TTGCTGCTGCTGCTGCTGCTGCTGCTGCTG	TTGCTGCTGCTGCTGCTGCTGCTGCTGCTGCTG	In_Frame_Ins	INS
	ZRSR2	X	15821890	G	A	Missense_Mutation	SNP

Supplementary Table 7: the number of ubiquitous and private CNVs

Patient	Common	HCC	iCCA
P1	17	410	550
P2	2	300	71
P3	42	183	152
P4	36	244	336
P5	0	121	205
P6	0	178	240
P7	5	90	182

Supplementary Table 8: The distribution of driver mutation genes in evolutionary tree

**Patient**	**Trunk**	**Brach (HCC)**	**Branch (iCCA)**
P1	TP53, PTPRC	USP28, MEF2A, LRIF1, GRZN2A, FGFR1 EML4, ARHGAP35, AKAP9	NF2, HUWE1, CDH1
P2	CARD11, CBLB, DMD, H3F3A, OLIG2, PTPN13 SPTAN1, ZFHX3	SND1, TCEA1	-
P3	CACNA1D, FOXP1, PDGFRA, SOS1, TP53 ZFP36L2	ERCC4, HOOK3, METAP1, PIK3CB PRPF8	ACKR3, ACVRZA
P4	FANCC, KAT6B, RBM10, RBM15, TP53	AFF3, QKI	MYO5A, NEB, SYNE1
P5	CIITA, JMJD1C, KALRN, MED13	5-seq, ACVR2A, MTOR, NFATC2, RUNX1T1	SYE1
P6	ANKRD11, ARID1B, ASPM, FBXO11, HUWE1, MET, PIK3R1, PTPN13, TP53, TP53BP1	AHDC1, APC, LMNA, NCOA4	ADCY1, GMPS, NIPBL
P7	BAP1, CDKN2A	CHN1, H3F3A	ACSL6

Supplementary Table 9: Mutation SMGs associated with stem cell and differentiation

#Gene	Variant classification	Indels	Variant classification	SNVs	Tot Muts	Sample Affect	Sample Percent (%)	Patient number	P-value CT	FDR CT
TP53	Frameshift	4	Missense	4	8	8	57.14	4	1.89E-18	3.63E-14
ACVR2A	_	0	Missense	2	2	2	14.29	2	0.001206077	0.092

ANO3	_	0	Missense	4	4	3	21.43	2	0.0000159	0.013
ARID1B	-	0	Missense	4	4	4	28.57	2	0.0000543	0.023
ASXL3	-	0	Missense/Nonsesne	3	3	3	21.43	3	0.000307308	0.046
BBS12	Frameshift	2	Missense	1	3	3	21.43	2	0.00000395	0.007
FCGBP	Frameshift	1	Missense	5	6	5	35.71	3	0.0000133	0.013
FREM1	-	0	Missense	4	4	4	28.57	2	0.0000357	0.02
GRM1	-	0	Missense/Nonsense	4	4	4	28.57	2	0.00000602	0.008
HGFAC	-	0	Missense	2	2	2	14.29	2	0.003517695	0.189
MORC1	-	0	Missense/Nonsense	4	4	4	28.57	2	0.00000248	0.005
MUC2	-	0	Missense	3	3	3	21.43	2	0.003351305	0.181
MUC4	-	0	Missense	5	5	2	14.29	2	0.000761952	0.069
NFATC3	-	0	Missense	4	4	4	28.57	2	0.0000047	0.008
RELN	-	0	Missense	3	3	3	21.43	2	0.00209337	0.129
TBC1D9	-	0	Missense	3	3	3	21.43	2	0.000107379	0.03
VCAN	-	0	Missense	3	3	3	21.43	2	0.00098821	0.082

Supplementary Table 10: Mutaion genes in the signaling pathways regulating pluripotency of stem cells

#Gene	Variant classification	Indels	Variant classification	SNVs	Tot Muts	Sample Affect	Sample Percent(%)	Sample
ACVR2A	_	0	Missense	2	2	2	14.29	P3, P5
FZD9	_	0	Missense	2	2	2	14.29	P5
ZFHX3	_	0	Missense	2	2	2	14.29	P5
PIK3R1	_	0	Missense	2	2	2	14.29	P6
WNT16	-	0	Missense	2	2	2	14.29	P4
HAND1	-	0	Missense	2	2	2	14.29	P5
PIK3CB	-	0	Missense	1	1	1	7.15	P3
ESX1	-	0	Missense	1	1	1	7.15	P6
DUSP9	-	0	Missense	1	1	1	7.15	P1
SKIL	-	0	Missense	1	1	1	7.15	P4
APC	-	0	Missense	1	1	1	7.15	P6
FGFR1	-	0	Missense	1	1	1	7.15	P1

Supplementary Table 11: Mutation genes in the Wnt and Notch Pathways

#Gene	Variat clsssifcation	Indels	Variant classifyion	SNVs	Tot Muts	Sample Affect	Sample Percent(%)	Sample	Pathway
TP53	Frameshift	4	Missense	4	8	8	57.14	P1, P3, P4, P6	Wnt
NFATC3	-	0	Missense	4	4	4	28.57	P1, P4	Wnt

FZD9	_	0	Missense	2	2	2	14.29	P5	Wnt
WNT16	_	0	Missense	2	2	2	14.29	P4	Wnt
NFATC2	_	0	Missense	1	1	1	7.15	P5	Wnt
APC	_	0	Missense	1	1	1	7.15	P6	Wnt
CTBP1	_	0	Missense	1	1	1	7.15	P1	Wnt
DKK1	_	0	Missense	1	1	1	7.15	P6	Wnt
VANGL1	_	0	Missense	1	1	1	7.15	P6	Wnt
CTBP2	_	0	Missense	1	1	1	7.15	P1	Wnt
JAG1	_	0	Nonsense	1	1	1	7.15	P6	Notch
NUMB	_	0	Missense	1	1	1	7.15	P4	Notch

Supplementary Table 12: Tumor purity and cellularity

Sample	Purity (Pathologist)	Purity (ABSOLUTE)	ploidy
C1	0.8	0.58	4.88
C2	0.8	0.8	5.38
C3	0.85	0.66	4.57
C4	0.8	0.75	5.24
C5	0.9	0.8	5.8
C6	0.9	0.5	3.13
C7	0.8	0.5	2.28
H1	0.9	0.7	5
H2	0.9	0.8	4.38
H3	0.85	0.5	2.6
H4	0.9	0.8	4.77
H5	0.9	0.66	4.08
H6	0.9	0.66	4.37
H7	0.9	0.66	3.78
