# Supplementary material for: Whole-exome sequencing reveals the origin and evolution of hepato-cholangiocarcinoma
Source: Nat Commun. 2018 Mar 1;9:894. doi: 10.1038/s41467-018-03276-y (PMC5832792; doi:10.1038/s41467-018-03276-y)
Supplement: Supplementary file 3 — Description of Additional Supplementary Files [file 41467_2018_3276_MOESM3_ESM.pdf]

Description of Additional Supplementary Files

File Name: Supplementary Data 1
Description: Information of 75 included liver cancer patients

File Name: Supplementary Data 2
Description: Distribution of nonsynonymous somatic mutations of 7 H-ChC patients; GT: genotype (homozygote:0/0; heterozygote: 0/1); AD: allelic depths; BQ: base quality; DP: depth; FA: allelic frequency; SS: mutation type (0: wild type; 1: germline mutation; 2: somatic mutation; 3: LOH; 4: post-transcriptional modification; 5 ： unknown information.

File Name: Supplementary Data 3
Description: The distribution of somatic CNVs
